# Beyond (Mis)Representation: Visuals in COVID-19 Misinformation

**DOI:** 10.1177/1940161220964780

**Published:** 2021-01

**Authors:** J. Scott Brennen, Felix M. Simon, Rasmus Kleis Nielsen

**Affiliations:** 1 University of Oxford, Oxford, UK

**Keywords:** COVID-19, coronavirus, misinformation, visual framing, social media, memes, fake news

## Abstract

This article provides one of the first analyses of visuals in misinformation concerning COVID-19. A mixed-methods analysis of ninety-six examples of visuals in misinformation rated false or misleading by independent professional fact-checkers from the first three months of 2020 identifies and examines six frames and three distinct functions of visuals in pieces of misinformation: how visuals illustrate and selectively emphasize arguments and claims, purport to present evidence for claims, and impersonate supposedly authoritative sources for claims. Notably, visuals in more than half of the pieces of misinformation analyzed explicitly serve as evidence for false claims, most of which are mislabelled rather than manipulated. While this analysis uncovered a small number of manipulated visuals, all were produced using simple tools; there were no examples of “deepfakes” or other artificial intelligence-based techniques. In recognizing the diverse functions of visuals in misinformation and drawing on recent literature on scientific visualization, this article demonstrates the value in both attending to visual content in misinformation and expanding our focus beyond a concern with only the representational aspects and functions of misinformation.

After the World Health Organization (WHO) named the “over-abundance of information” around the SARS-CoV-2 outbreak and response an “infodemic” in early February 2020 (Thomas 2020; WHO 2020), journalistic, academic, and public interest in the spread of COVID-19-related dis- and misinformation skyrocketed. At the same time, scholars of misinformation have increasingly recognized that visuals not only are regularly included in false content, but that they may play a key role in mediating the effectiveness and impact of misinformation. Despite this, very little published research has focused specifically on visuals in COVID-19-related misinformation.

This article provides one of the first analyses of visuals in pieces of misinformation concerning COVID-19. We present a mixed-methods analysis of the visuals in ninety-six pieces of COVID-19 misinformation rated false or misleading by independent professional fact-checkers and produced in the first three months of 2020. Conceptually, we follow Vraga and Bode’s (2020) definition of misinformation as “information considered incorrect based on the best available evidence from relevant experts at the time,” and in our research design, we rely on the work of independent professional fact-checkers to identify the material we then analyze. Notably, we do not distinguish between disinformation—the “*intentional* spread of false information, for example, driven by political strategies [. . .] where the communicator distributes incorrect information to achieve a certain (political) goal” (Hameleers, Powell, et al. 2020: 282–83)—and misinformation, false or misleading information spread unintentionally (Wardle 2017, para. 1). As we are unable to establish the intent of those creating and/or spreading the pieces we analyze, we use the term “misinformation” throughout.

Examining both textual and visual elements, our analysis identifies six distinct frames within pieces of COVID-19 misinformation: authoritative agency, virulence, medical efficacy, intolerance, prophecy, and satire. We also identify and discuss three broad functions that visuals play within pieces of misinformation: illustrating and selectively emphasizing elements of misinformation, purporting to present evidence for claims, and impersonating authoritative institutions. While illustrating and selectively emphasizing elements of content, visuals often directly help construct or support frames; visuals also contribute to frames while serving as evidence and impersonating authorities.

In identifying and analyzing both the frames that visuals help construct as well as the diverse functions visuals play within pieces of misinformation, this article demonstrates the value in attending to visuals in misinformation—a topic that remains under researched. At the same time, scholars, journalists, fact-checkers, and policymakers have often approached misinformation through a representational paradigm focused somewhat narrowly on whether visuals and texts represent or contradict the best available evidence. While the representational aspects of misinformation are deeply important, research, both in visual studies and on the role of visuals in scientific research (Daston 2014), demonstrates that some visuals not only represent, they also constitute and coproduce objects and authority. In particular, photographs and videos do so in part through their assumed indexicality, a “physical connection” (Peirce 1955: 106) to the objects they depict. Without discounting the importance of attending to the representational dimensions of misinformation, we argue that to advance understanding of the role of visuals in COVID-19 misinformation and misinformation more broadly it is important to look beyond the representational capacities of visuals. This is powerfully illustrated by our finding that while the visuals in more than half of the pieces of misinformation in our sample are presented as evidence for false claims, the visuals themselves are mostly mislabelled or used in other misleading ways, but are not, when considered in isolation, false or manipulated.

## COVID-19 Misinformation

While mis- and disinformation about health topics is neither new nor unique to the coronavirus pandemic, misinformation concerning COVID-19 has attracted a great deal of attention. Much of the initial research has focused on the types, origins, and spread (Brennen et al. 2020) as well as possible effects of false information and conspiracies around the coronavirus and the pandemic (Freeman et al. 2020). Academic, industry, and journalistic accounts have examined the propagation of COVID-19 disinformation by state actors (Swan 2020), the prevalence of COVID-19 conspiracy theories (Freeman et al. 2020; Uscinski et al. 2020), the spread of misinformation on social media on platforms such as Facebook and Twitter (Hollowood and Mostrous 2020), as well as interventions to counteract misinformation on social media (Pennycook et al. 2020).

Employing a quantitative content analysis, Brennen et al. (2020) found a predominance of reconfigured content, with a majority of misleading or false claims focusing on the actions or policies of public authorities, including government and international bodies like the WHO or the United Nations. Surveying Internet users in six countries in the spring of 2020, Nielsen et al. report that a high number of citizens say that they came across false or misleading information about COVID-19, in particular on social media (Nielsen et al. 2020), a finding backed by Hameleers, van der Meer, et al. (2020) in a study of four countries.

To the best of our knowledge, however, no study to date has viewed COVID-19 misinformation through a visual lens, focusing explicitly on the visual content in coronavirus misinformation, its function, and its characteristics.

### Visual Misinformation

Despite the proliferation of scholarship on misinformation over the last several years, and the broader observation that we live in a “visual culture” where much signification is carried out through visuals (Evans and Hall 1999: 2), there remain few studies of visuals in mis- or disinformation. Recent work by Hemsley and Snyder, for instance, provides a framework of how visual artifacts contribute to misinformation (Hemsley and Snyder 2018) while Hameleers, Powell, et al. (2020) investigate the credibility of textual versus multimodal (text-plus-visual) disinformation, finding that multimodal disinformation is considered slightly more credible than textual disinformation. Despite these examples, broader studies of visuals in misinformation remain few and far between. A likely explanation for the dearth of studies is that collecting, storing, and analyzing visual content, especially at scale, presents significant practical and methodological challenges. Some have approached these challenges by choosing to analyze image metrics or metadata rather than parsing visual content itself. Notably, a number of studies have used this approach to assess the amount or prominence of misinformation or low-quality information on YouTube (cf. M. N. Hussain et al. 2018).

Fewer studies have analyzed visual misinformation content itself. Some have used automated image analysis, such as Google Cloud Vision (Zannettou et al. 2020) to convert images into (textual) topics. Others have employed human coding of videos. Garimella and Eckles (2020) found that a majority of visual misinformation in public Indian political WhatsApp groups consisted of images misidentified or taken out of context. Many have looked at the quality of videos prioritized by the YouTube search algorithm. Findings have varied widely. While some have found conspiracy theories, climate denial, and extremist content common on YouTube (Allgaier 2019; Lewis 2018), others have found that YouTube’s algorithms are promoting mainstream content (Bounegru et al. 2020; Ledwich and Zaitsev 2020). More relevant here, Marchal et al. (2020) found YouTube’s search algorithm prioritizing COVID-19-related content from legitimate institutional sources.

### Visual Framing

While studies of visual misinformation are limited, scholars across fields employ a variety of forms of visual analysis. Visual framing analysis is becoming a prominent form of visual analysis. Coleman (2010) defines visual framing as a form of framing analysis. She draws on Entman’s (1993: 52) well-known definition of framing to define visual framing as the “selection of one view, scene, or angle when making the image, cropping, editing or selecting it” (Coleman 2010: 237) that “selects some aspects of a perceived reality and makes them more salient in a communicating text, in such a way as to promote a particular problem definition, causal interpretation, moral evaluation, and/or treatment recommendation” (p. 235). Importantly, visual material often occurs along with text. While the frames communicated in text and visuals do not always align, Coleman concludes that nonetheless, “it is never enough to study framing in one mode of communication and not the other” (Coleman 2010: 237). Instead, we must adopt a multimodal framing approach that acknowledges and investigates how text and visuals align, conflict, and/or are intertwined. That is the approach we adopt here.

Empirical visual framing studies have looked at a wide range of news, political, scientific, energy, and climate-related content and have adopted a range of approaches (Bucy et al. 2020; Grabe and Bucy 2009; Krause and Bucy 2018; Wessler et al. 2016). As Bock (2020) notes, this diversity has meant that “visual framing suffers from the same theoretical cloudiness as framing generally” (p. 4). Addressing the “theoretical cloudiness” of visual framing, Bock (2020) offers a three-part model that aims to push research beyond “literal depictions and descriptions” to realize “the paradigm’s explanatory and critical potential” (p. 1) by recognizing the work that visual framing can do in the wider world, well beyond merely representing it.

### Beyond Representation

The imperative to systematically attend to the effects and wider implications of visuals is fundamental to the efforts to reconceptualize (visual) framing described above. Recognizing the wider roles that visuals serve has been key to visual studies and visual culture research (Mitchel 2005). For some, this imperative is rooted in the work of the philosopher C.S Peirce, whose influential theory of signs holds that while *symbols* have no necessary connection to their referent, and *icons* bear a visual similarity, *indices* have a “physical connection” and a causal relationship to their referents (Peirce 1955: 106). For the past 150 years, scholars have argued for and against the indexicality of photographs, and the ways in which “photographs come with an implicit guarantee of being closer to the truth than other forms of communication are” (Messaris and Abraham 2001: 217). Yet, acknowledging the indexicality of visuals emphasizes how materiality mediates the diverse functions visuals play across social life.

Recent scholarship on visualizations in science also recognizes the role that visuals may play across different domains of social life. Given how much of it purports to deal with scientific issues, this literature is particularly relevant for understanding the role of visuals in COVID-19 misinformation. Situating visuals within scientific practice involves attempting “to think about images beyond representation” (Daston 2014: 320): to recognize the wider universe of connections and practices in which visuals participate. Moving beyond representation means shifting from an epistemological treatment of visuals to an ontological one. Such a move would “collapse the distance between presentation and representation: the image *is* the presentation” (Daston 2014: 320–21). In part, this is to acknowledge that visuals are performative (MacKenzie 2006), that they don’t just represent, they also constitute or coproduce (Jasanoff 2004) the objects we encounter in the world. Understanding the complex roles that visualizations play in scientific practice can help us move beyond a narrow concern with the gap between visuals and the reality they purport to represent and see the diverse set of practices and relations that visuals produce and are produced by. As one of the “working object[s] of science” (Daston 2014: 320), and of misinformation (Garimella and Eckles 2020), visuals matter because of what they help constitute and facilitate.

Much of the research on misinformation over the past several years has directly addressed how misinformation does not represent reality, how (mis)representations circulate, and whether they are convincing. These are fundamentally important questions. However, the literature on scientific visualizations (as well as that on visual culture) suggests that we should *also* recognize ways in which pieces of misinformation can help constitute objects and things in the world, how they are embedded in social practice, and how they mediate authority and power. Given this, we ask the following research questions:

**Research Question 1 (RQ1):** What frames commonly appear in COVID-19-related misinformation containing visuals?
**Research Question 2 (RQ2):** How does visual material help constitute the frames found in pieces of misinformation?
**Research Question 3 (RQ3):** What functions does visual material serve in pieces of misinformation?

## Method

To address these three research questions, we completed a mixed-methods analysis of a corpus of pieces of COVID-19 misinformation identified through existing fact-checking articles from January 2020 through March 2020. First, to gather a workable set of content, we randomly sampled 18 percent of a corpus of 1,253 fact-checks of pieces rated false or misleading by international fact-checkers such as Associated Press, Snopes.com, Full Fact, or BOOM and compiled by the International Fact-Checking Network (IFCN)^1^ and Google Fact Checking Tools^2^ and gathered by First Draft (See Table 1 for a list of the fact-checkers). We replaced duplicate entries with additional randomly selected entries. This corpus of 225 pieces of misinformation supplied a previous study (Brennen et al. 2020). For this article, we selected all the pieces within this 225-item content sample that contained an image or video. After removing a handful of pieces that were no longer accessible, we were left with a corpus of 96 pieces of COVID-19 misinformation containing one or more visuals, which were used for the analysis in this article.

**Table 1.** List of Fact-Checking Organizations.

Number	Fact-checker	Country
1	AAP Factcheck	AUS
2	Times of India	IND
3	India Today	IND
4	BOOM	IND
5	PolitiFact	USA
6	Vera Files	PHL
7	FactCheck.org	USA
8	AFP Indonesia	IDN
9	AFP South Africa	ZAF
10	Snopes.com	USA
11	FactCrescendo	SRL
12	AFP Philippines	PHL
13	Newschecker	IND
14	Truthorfiction.com	USA
15	The Logical Indian	IND
16	Rappler	PHL
17	Check Your Fact	USA
18	AFP India	IND
19	Full Fact	UK
20	Factly	IND
21	Dubawa	GHA
22	Alt News	IND
23	Annie Lab at JMSC	HKG
24	Polygraph	USA
25	AFP Singapore	SGP
26	LeadStories	USA
27	AFP USA	USA

*Note.* Table 1 contains the list of fact-checking organizations from which the visual pieces of COVID-19 misinformation used in our analysis derive.

All visuals were coded by two coders based on a predefined coding scheme. An initial codebook containing a range of variables addressing both visual composition and visual function, as well as a typology of frames, was produced by drawing on existing literature on visual framing and health communication. The codebook was tested and refined over two initial rounds of test coding, whereby the coders independently coded a random selection of 20 visuals in both rounds. After each round, the results were compared and discussed and the codebook refined. Once the codebook was set, both coders recoded all 96 pieces of content. Differences in coding were discussed and resolved until consensus was reached. After coding examples of each frame, we then undertook a more interpretative visual framing analysis to better understand how exactly visuals constructed frames, the way frames encode ideologies (Bock 2020), how text and images relate, and the roles that visuals play within pieces of COVID-related content.

Importantly, this sample only contains misinformation found and selected by international fact-checkers and compiled by IFCN and Google Fact Check Tools. It is therefore not representative of the entire landscape of COVID-19 misinformation. Fact-checkers decide independently which claims to check, often based on a mix of active monitoring of other news media and online sources, as well as audience submissions. Claims circulating in messaging apps and private groups on social media platforms, for instance, are likely underrepresented in international fact-checks, given that they usually circulate in places that are hard to access for fact-checkers. Similarly, fact-checkers must make choices of how to use limited time and resources and will often try to focus their efforts on what they regard as the most prominent, important, or potentially harmful misinformation they are aware of. While there is no guarantee that fact-checkers are assessing a representative sample of misinformation circulating, our convenience sample offers insight into the types of visuals found within some important examples of misinformation about COVID-19 in the early stages of the pandemic.

## Findings

### Frames

Addressing RQ1, we identified six main frames in the corpus of misinformation containing visuals: *authoritative agency, virulence, medical efficacy, intolerance, prophecy*, and *satire.* These frames are supported by both the visuals and the text in the pieces of content in our sample. Notably, some pieces of misinformation contained multiple frames, and so the percentages add up to more than 100. Table 2 provides the prevalence of each frame within the sample. Below we investigate in more detail how visuals help constitute each frame, identifying three specific functions that visuals perform within pieces of misinformation: visuals illustrate and selectively emphasize elements of claims, they provide purported evidence for claims, and they impersonate authorities. Before discussing these functions in depth, we introduce the six frames observed in the corpus in more detail.

**Table 2.** The Most Common Frames.

	Frame	Description	Example	Prevalence (%)
1	Authoritative Agency	Valanced claims about actions of public authorities	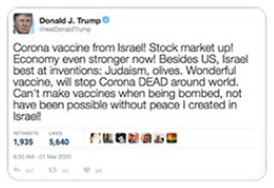	40
2	Virulence	Over- or understates the spread of the virus, or claims that the disease is not real	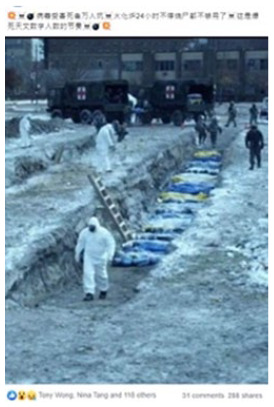	33
3	Medical Efficacy	Offers medical information, highlights tests, vaccines, equipment, suggests that there exist cures, treatments, or preventatives for the virus	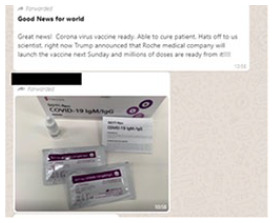	29
4	Intolerance	Expresses racism, xenophobia, sexism, etc.	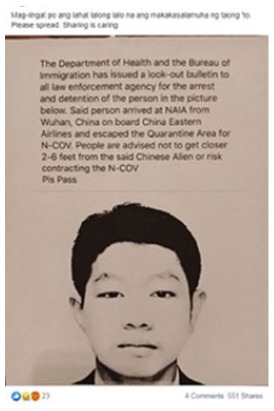	15
5	Prophecy	Suggests virus was previously predicted	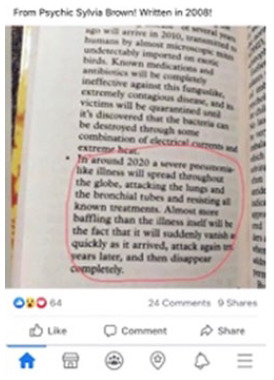	10
6	Satire	Satirical or humorous content	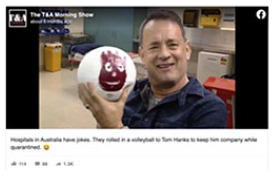	6

*Note.* Table 2 identifies the most common frames found within the corpus and provides each frame’s prevalence in the sample. Some pieces of misinformation were coded as communicating more than one frame.

#### Authoritative agency

The most common visual frame revolves around the actions or agency of public authorities such as ministries or the WHO, occurring in 40 percent of all pieces of false or misleading content. We observed, however, a range of variations of this frame: some pieces of misinformation claim public authorities are doing more than they are; others that they are doing less. Some pieces of misinformation celebrate the actions public authorities are (or are not) supposedly taking, while others lament them. But what defines this broad frame are valanced claims made about what institutions are or are not accomplishing: positive or negative emphasis of the purported actions of public authorities. As discussed in more detail below, visuals help establish or support these frames in several different ways. Most notably, however, visuals can help lend authority to a piece of misinformation by impersonating the markings of real institutions (see Figures 7 and 8), or by providing purported evidence for a claim *about* what that authority is doing.

#### Virulence

The second most common frame is virulence, present in 33 percent of all cases. The vast majority of examples of this frame claim that the spread of the virus was *worse* than it really was. Many of the pieces of misinformation with this frame claim that the virus was spreading in ways or in places it was not (see 2 in Table 2). A small number of pieces of misinformation claim that the virus and the disease are not real—and therefore that the virus is *less* virulent than it actually is. In many of the examples of content with this frame, the visuals purport to offer evidence for the supposed spread of the virus.

#### Medical efficacy

Almost a third of the pieces of visual misinformation, 29 percent, contain a frame we call medical efficacy. Most examples of this frame are positive or hopeful, suggesting that there exist cures, treatments, or preventatives for the virus (see 3 in Table 2). Visuals in examples of misinformation communicating this frame serve all three functions discussed below: selectively emphasizing, serving as evidence, and impersonating authority.

#### Intolerance

Some 15 percent of the visual misinformation analyzed contained racist, xenophobic, or extreme partisan elements, that establish a frame we broadly call “intolerance” (see 4 in Table 2). Visuals in the corpus not only selectively emphasize elements to promote this frame, they also provide purported evidence of the nefarious deeds of targeted populations. It is the false or misleading element to these pieces that means they are not just racist or intolerant, but also misinformation.

#### Prophecy

About one in ten posts (10 percent) claim that someone predicted the emergence of the novel coronavirus. There is a reasonable argument that some of these claims should not be considered misinformation. For example, one very popular social media post shows an image of a book published in 2008 by the psychic Sylvia Browne in which she predicted the emergence of a “severe pneumonia-like illness . . . around 2020” (see 5 in Table 2). Brown did in fact publish a book in 2008 with this claim. However, we follow fact-checkers in labeling these claims as misinformation. In addressing these sorts of claims, fact-checkers either demonstrate that these predictions were not completely accurate or address the frequent implication that if the virus was predicted, it was necessarily planned or manufactured in a laboratory. Our corpus does include one example where a screenshot from an episode of the Simpsons was doctored to make it seem as though it had predicted the virus. Notably, in all of the examples containing this frame, visuals serve as evidence documenting the predictions made well before the virus emerged.

#### Satire

Finally, 6 percent of the visual content analyzed was coded as satire. Like those in the previous category, there are arguments that satire should not be considered misinformation. It is included here for two reasons. First, despite being intended as satire, some people may still misinterpret such posts as serious. Second, fact-checkers have labeled these claims as false or misleading. As discussed above, we rely on fact-checkers to evaluate the facticity of content. In one example (see 6 in Table 2) originally from *The Betoota Advocate*, which the fact-checker CheckYourFact describes as “an Australian satire website,” the creator(s) photoshopped an image of Tom Hanks with a volleyball in a hospital room along with the text “Hospitals in Australia have jokes. They rolled in a volleyball to Tom Hanks to keep him company while quarantined. ” The fact-checker labeled this as false, noting that despite being intended as a joke or satire, the image was digitally manipulated and that some social media users “circulated that photo as being real” (Schakohl 2020).

#### Functions

Addressing RQ2 and RQ3, we identify three distinct functions that visuals serve within COVID-related misinformation. We coded these three functions as mutually exclusive (see Table 3). Each of these functions is discussed in turn below. It is important to note that these are not the only functions visuals serve in COVID misinformation. However, taking these as broadly inclusive types we were able to categorize every case in the corpus into one of these three functions. Importantly, in emphasizing certain elements, serving as evidence, or impersonating authorities, visuals in the sample *also* contribute to the six frames discussed above.

**Table 3.** Frequencies of Each Function of Visuals in Misinformation.

Function	Frequency (%)
Illustrating and selectively emphasizing	39
Serving as evidence	52
Impersonating authorities	9

*Note.* Table 3 provides frequencies of each function identified in the sample. Percentages refer to the percent of the total sample serving each function. These are coded as mutually exclusive.

### Illustrating and Selectively Emphasizing

In almost 40 percent (39 percent) of all the pieces of misinformation in our corpus, visuals are broadly used to illustrate some element of the claim being made. In doing so, they selectively emphasize certain features of the claim while ignoring others. Looking across the corpus, we found evidence that this selective emphasis was utilized to achieve a range of different ends.

In some examples, the images present a straightforward illustration of key aspects of the claim. While we are not able to know the intention of misinformation producers, it is possible that some images are used simply to draw more attention to claims to aid their circulation across social platforms. Vaccari and Chadwick (2020) observe that “visuals enhance the transmission of information by helping people to establish and retrieve memories [. . . ]” and that “individuals process visual information more directly and with less effort than verbal information” (p. 2). For example, one post claims smoking may prevent COVID-19 infection. It includes what appears to be a stock image of a cigarette. This visual in isolation is neither false nor misleading (it *is* an image of a cigarette), but it is used to illustrate a piece of false and misleading misinformation.

**Figure 1.**
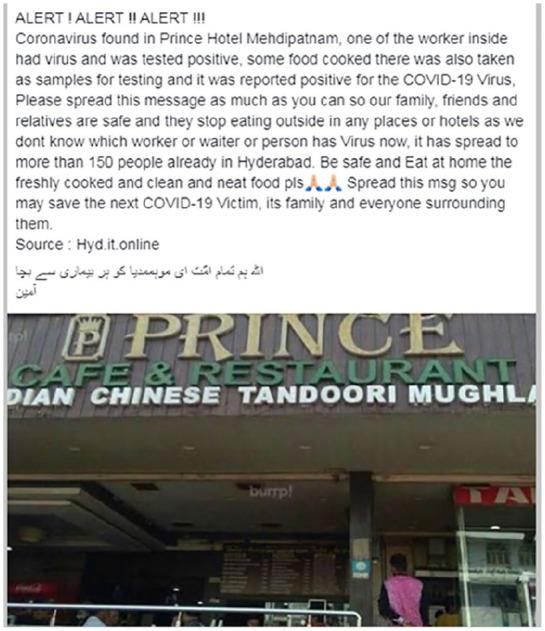
Facebook post falsely accusing a restaurant of being the site of a COVID-19 outbreak.

Selectively illustrating or emphasizing content can also serve more specific ends. For example, one Facebook post claims that a worker in a restaurant in Hyderabad, India, had tested positive for and potentially spread the virus. The post was accompanied by a picture of the restaurant (Figure 1). Here, the visual does not serve as evidence for the claim made in the piece of misinformation, nor does it purport to support a claim the speaker is from some authoritative institution. However, by prominently displaying the name of the restaurant, the visual makes it more likely readers remember which restaurant is implicated. Again, the photo appears to be real and in itself is an accurate representation of a restaurant, but it is used to make a false claim.

**Figure 2.**
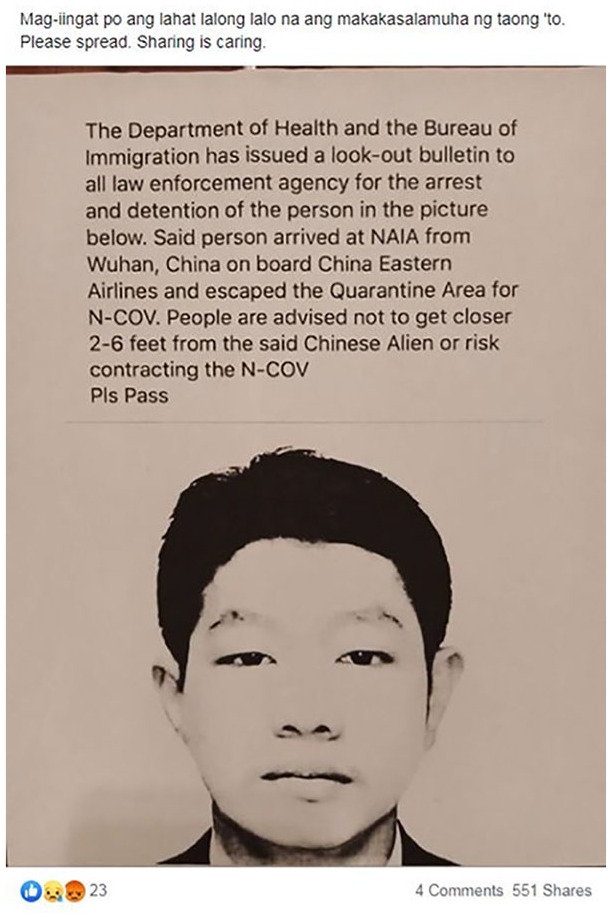
Facebook post falsely claiming a Chinese national was spreading SAR-CoV-2 in the Philippines.

Other visuals appear intended to provoke an emotional response in audiences. For example, one post includes a visual that resembles a mug shot or wanted poster of a man from China falsely accused of spreading the virus (Figure 2). The manner in which the image is presented helps to assert the claim that he is a danger to public health in a very particular and racialized fashion.

**Figure 3.**
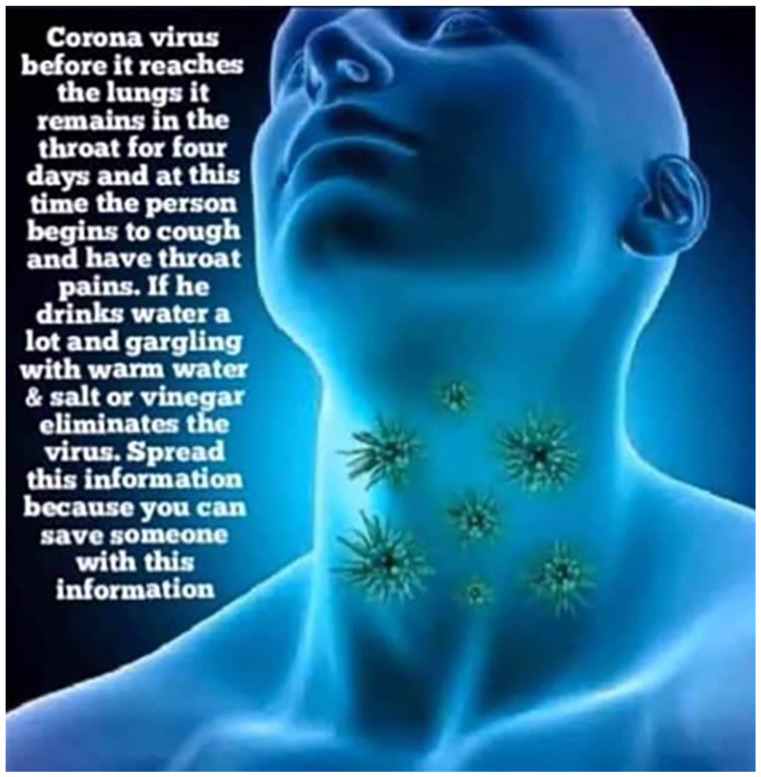
Facebook post falsely claiming that drinking or gargling warm salt water can prevent COVID-19 infection.

Another post uses a computer animation to illustrate a false claim that drinking water or gargling saltwater or vinegar can prevent infection. By illustrating the (false) mechanism through which drinking water/gargling supposedly prevents invention, the visual helps to lend medical authority to the claim, supporting the “medical efficacy” frame (Figure 3).

### Serving as Evidence

In more than half (52 percent) of the number of pieces of false or misleading content analyzed for this study, visuals explicitly serve as evidence for claims. Visuals-as-evidence in the sample consist of three types of content. The most common form in our sample is what Wardle (2019) has called “false context,” in which an image or video segment is labeled or described as being something it is not (35 percent of all content).^3^ Importantly, in these examples, the images (or videos) are not edited or manipulated; they are simply misidentified. Many other studies have similarly identified “false context” as a prominent form of misinformation. For example, Garimella and Eckles (2020) estimated that 34 percent of the visual misinformation in their sample of public Indian political WhatsApp groups took this form.

Second, about 10 percent of all pieces of content in the sample were identified by fact-checkers as being manipulated. Images were modified, or video was selectively edited to suggest something that was false or misleading. Only in this small subset of cases can the visuals themselves in isolation be said to be false. Manipulated content serves an evidentiary role similar to “false context,” in which the visual content is used to directly “prove” or support the claim. Of the ten pieces of manipulated content in the sample, all involve either relatively simple video editing or photo manipulation. We saw no examples that used more complicated or powerful manipulation techniques or software. Most notably, we saw no examples of “deepfakes” or other use of artificial intelligence (AI)-based tools.

Finally, a small number of pieces of content in the sample (6 percent) serve as evidence for claims but represent neither false context nor manipulated content. For example, as discussed above, one post offers a photograph of a page of a book (see 5 in Table 2), as evidence that Sylvia Browne predicted the pandemic in 2008. Here, it is the post that is misleading, not the visual or how the visual is identified.

**Figure 4.**
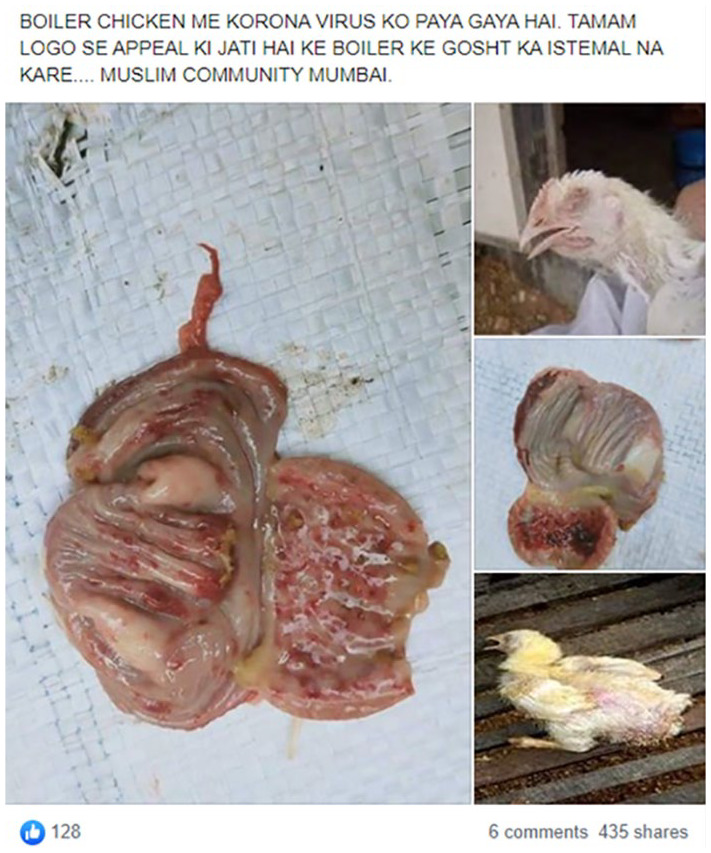
Facebook post purporting to show a chicken infected with COVID-19.

In serving as evidence for claims, visuals directly support the facticity of claims. In doing so, images function within the “fixation of evidence”: the ways that visuals come to serve as evidence to support beliefs (Amann and Cetina 1988). While the evidentiary function of these visuals certainly involves how they represent the world, it also speaks to something else—something that draws more on the assumed “indexicality” of photographs and video—their *direct connection* to the world. For example, one visual in our sample purports to show a chicken that has been infected with COVID-19 and is contagious to humans (Figure 4). Yet, while the post claims that the visual is direct evidence, that claim still hinges on trusting the reported identity of what is depicted. Few of us are able to know if the diseased chicken shown has been infected with COVID-19. Instead, we have to trust the text in the post that this is the case—or the fact-checkers that it is *not*.

**Figure 5.**
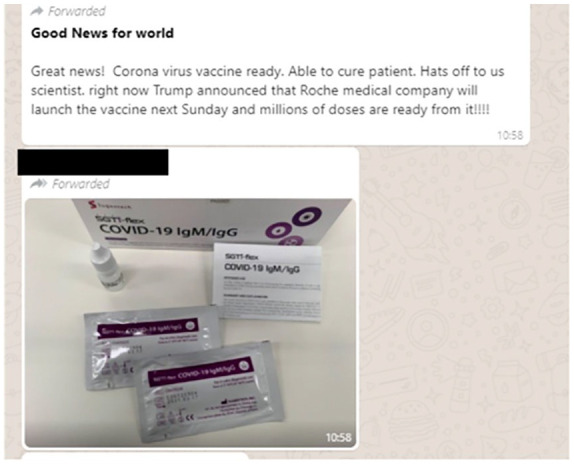
WhatsApp messsage falsely claiming to show a new COVID-19 vaccine.

This image not only (supposedly) serves as evidence for this diseased and infectious chicken, it also offers a revision in our understanding of the virus and the danger it poses. This example highlights the way that in serving as evidence for claims, visuals help establish new or reformed objects—often those related to the virus in one way or another. For example, along with a claim that Donald Trump was set to announce a new vaccine produced by the Swiss pharmaceutical company Roche, the WhatsApp post in Figure 5 includes an image it identifies as the vaccine. In reality, the image shows a COVID-19 testing kit. Two additional pieces of misinformation show images from medical textbooks to suggest that the (new) coronavirus is simply the “common cold.” In this way, the photographic “evidence” is intended to reconstitute the virus as a non-serious threat.

**Figure 6.**
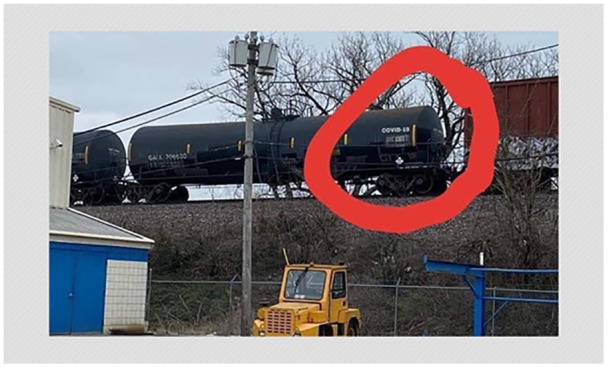
Manipulated image purporting to show a railtanker transporting SARS-CoV-2.

In some instances, the visuals are leveraged as evidence of broader phenomena. In one image shared on Facebook (Figure 6), someone photoshopped “COVID-19” on a rail tanker moving across the countryside. While the manipulated image offers evidence of the rail tanker, comments accompanying the post on Facebook attest that it also is meant to serve as evidence that the pandemic is part of a large conspiracy in which the virus has been manufactured and is being transported across countries.

In serving as “evidence” for a global conspiracy to manufacture and spread COVID-19, the manipulated image of the tanker also helps construct an example of the “authoritative agency” frame described above. The conspiracy supposedly involves governments or international institutions that wield global power. This claim not only mischaracterizes much of what we know about how the virus originated and spread but also widely overestimates the capacity of world governments and their purported malevolence in relation to the virus.

In addition to serving as evidence for claims, many visuals also support or construct the frames described above. As it presents supposed evidence that COVID-19 can infect humans through diseased meat, the stark foulness of the diseased chicken image in Figure 4 illustrates in sharp detail the presumed virulence of the virus. Amid what is clearly a partisan post, meant to support Donald Trump’s handling the of the pandemic, the image in Figure 5 of the misrepresented test kit helps frame the post as a clear medical advancement.

### Impersonating Authorities

**Figure 7.**
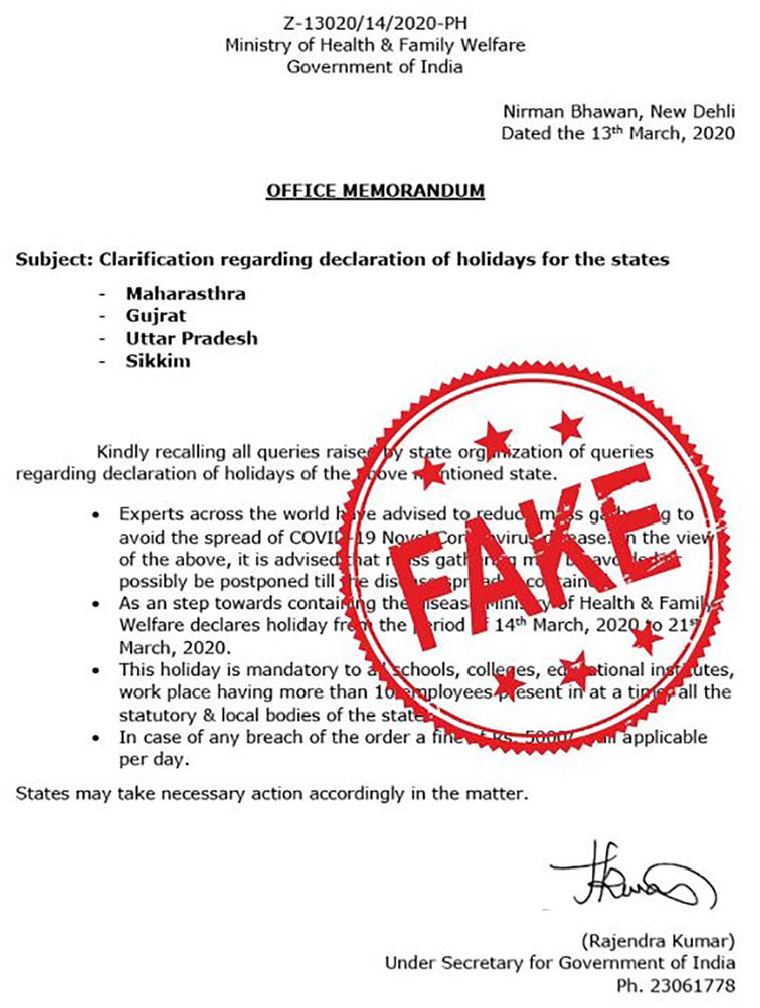
Counterfeit document purportedly from the Indian Ministry of Health and Family Welfare declaring a compulsory holiday in response to COVID-19.

Similar to the way that visuals can serve as evidence for claims made in pieces of misinformation, some visuals (9 percent) are used by producers of misinformation to impersonate authorities, such as governments or international health authorities. Visuals functioning in this way constitute what Wardle (2019) has labeled “imposter content.” For example, a counterfeit document from March 2020 (falsely) claims that the Indian Ministry of Health and Family Welfare had declared a compulsory holiday for several states due to COVID-19 (Figure 7).

The visual mimics the hallmarks of the Indian government using the typical format and language of official documents, including seals and logos. In effect, it takes advantage of the fact that people assess the credibility of information using various formatting and stylistic cues, including the false bureaucratic context and presentation of the information (Mercier 2020: 20).

**Figure 8.**
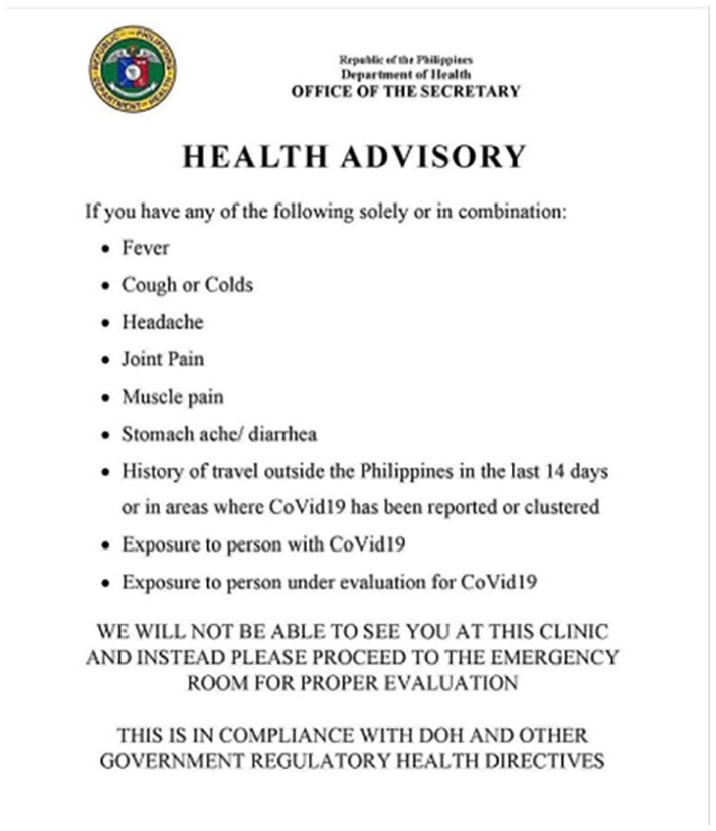
Counterfit document purportedly from the Philippine Department of Health advising that people infected with COVID-19 would be turned away from medical clinics.

Another example falsely claims that the Philippine Department of Health had issued orders that people infected with COVID-19 would be turned away from medical clinics (Figure 8). Again, the document deploys the visual markings of an official government document (or what people imagine such a document to look like), carrying the logo and letterhead of the Philippine Department of Health.

While allowing misinformation producers to impersonate authorities, these visuals also reinforce the frames identified in this analysis. Most commonly, the use of such visuals helps circulated misinformation communicate the “authoritative agency” frame. As seen in these examples, when mimicking authorities, posts claim that authorities are taking actions they are not—thereby overestimating the capacity and agency of institutions.

## Discussion

This study provides a starting point for the investigation of visuals in COVID-19 misinformation. Inquiring into the role that visuals play in pieces of false or misleading content based on a convenience sample of fact-checked misinformation, we identify three distinct functions of visuals in coronavirus misinformation: illustration and selective emphasis, serving as evidence, and impersonating authorities. As shown, in serving each of these functions, visuals also help constitute and communicate a series of frames that are false or misleading in different ways.

Given that our study is based on a small convenience sample of misinformation containing visuals, we cannot make general claims about the wider landscape of false or misleading coronavirus information. We are also not able to assess the intentions behind the use of visuals in misinformation, nor are we able to make claims about the reach of the examples in question. And while we consider how misinformation attempts to achieve certain ends or functions, on the basis of content analysis alone we are unable to assess the effects that these frames ultimately have on different audiences. That said, this analysis offers some initial insight into the frames found in coronavirus misinformation and the functions that visuals can serve. We hope this early insight will serve as a starting point for future studies and help advance understanding of the role of visuals in misinformation more broadly.

This study supports existing evidence that much of the misinformation circulating about COVID-19 contains visual elements (Brennen et al. 2020), as does misinformation more generally (Garimella and Eckles 2020). Amid a wider recognition that we need to take visual elements of news content seriously (see, e.g., Grabe and Bucy 2009; Schill 2012), we should also recognize that visuals play important roles within misinformation. Further research should investigate whether visuals in misinformation have an effect on the perceived credibility of and trust in the message presented; in other words, if there is a higher likelihood that audiences accept misinformation as true or believable if it is (co)presented with a visual. Future studies might also look into whether misinformation accompanied by visuals, packaged as visual memes, or forwarded simply in visual form travels more widely on social media than misinformation without. Finally, we hope further studies will look at the role of visuals in misinformation across different contexts (country-to-country, platform-to-platform) and topics (health, politics, science, etc.).

We have also found that manipulated content is rare in this corpus of misinformation. Every example of manipulated content in our sample employed simple photo or video editing techniques. We saw no examples of deepfakes or other AI-based manipulations. This is notable given the frequent concern expressed in both scholarly and public discussion about the threat deepfakes pose. While deepfakes may become more common over time, our findings suggest that, at least for now, misinformation producers are employing simpler means of producing false or misleading content. This finding also supports Paris and Donovan’s (2019) claim that successfully deceptive media are often “cheap fakes,” not necessarily requiring advanced processing technologies.

Our findings also suggest that while visuals play an important role in illustrating or representing aspects of false claims, they can serve additional functions. Most notably, we found that visuals were used as false evidence for claims, helping to establish objects as specific as a diseased chicken or as diffuse as a global conspiracy. Scholars would be well served by attending to the ways that visuals—whether taken out of context or manipulated—can work to ground and support false or misleading claims. There is reason to suspect that this sort of evidentiary support can bring into being claims that take on a life of their own—that move and grow and build new constituting networks of support (see Latour 1986). While it may be too early to tell how COVID-19 misinformation plays out in terms of its public health consequences, the example of the anti-vaccine movement is instructive. Even after the original 1998 *Lancet* article suggesting a link between vaccines and autism was discredited and retracted, the movement has persisted and likely even grown wider (A. Hussain et al. 2018; Schmidt et al. 2018). While fact-checking is broadly effective in countering false beliefs (Walter et al. 2020; Wood and Porter 2019), it may still have limited efficacy in stopping some of the particularly pernicious effects of misinformation, especially around misappropriated visual material.

This study also observed visuals employed with the aim of enabling providers of misinformation to impersonate institutions. While not directly supplying evidence for a claim, here visuals are meant to provide legitimacy or authority to claims. In light of the finding that the most prevalent frame observed concerns “authoritative agency,” these dynamics point to a need to further interrogate the relationships between visuals in misinformation and authority or power. Of course, disentangling the ways in which visual framing is implicated in complicated power dynamics has become a central goal of many strands of visual analysis, including visual framing (Bock 2020). At a minimum, our findings suggest that visuals play more complicated roles in conveying COVID-19 misinformation than merely depicting everyday objects and exaggerating their importance. Instead, we see visuals enfolded in far larger questions of politics and power, such as the authority of the United Nations or U.S. Centers for Disease Control and Prevention, the rights of immigrants, or geopolitical conflict between India and China. In much of the material we analyzed, the visuals themselves were not in isolation false or misleading representations of reality, nor were they fabricated or, in most cases, manipulated. Instead, their role goes beyond representation narrowly articulated and is largely illustrative, performative, and communicative.

In recognizing that visuals employed in the service of COVID-19 misinformation not only serve to illustrate and help frame misleading understandings about the virus, but also work to establish the authority and facticity of claims, our findings suggest the need for scholars of misinformation to recognize the range of functions that visuals can play. While establishing authority and facticity requires the representational capacities of visuals, these functions *also* speak to something beyond representation. In these instances, visuals not only represent the world, they also help construct or coproduce the claims made and attempt to bring into being objects and authorities, such as supposedly infected chickens, global conspiracies, or a harmless virus. Scholars of visual communication have long recognized the diverse roles visuals can play. Yet scholars of misinformation have not always considered the wider functions that misinformation serves beyond its representational capacity. Although some have begun to investigate these questions (e.g., Marwick 2018; Polletta and Callahan 2017; Vaccari and Chadwick 2020), our findings suggest scholarship should think more broadly about the ways that misinformation may act on audiences. Given the increasing popularity of visual-based social media platforms such as YouTube, Instagram, and TikTok (especially among younger audiences) for news and information consumption, it is paramount that we work to better understand the diverse roles and effects visuals can have within the larger landscape of misinformation.

## Notes

1. https://www.poynter.org/ifcn/.

2. https://toolbox.google.com/factcheck/explorer.

3. Interestingly, there were ten pieces of content (10 percent) that were false context but served to illustrate or emphasize rather than provide evidence for claims.
